# Associations of immune cell homing gene signatures and infiltrates of lymphocyte subsets in human melanomas: discordance with CD163^+^ myeloid cell infiltrates

**DOI:** 10.1186/s12967-021-03044-5

**Published:** 2021-08-28

**Authors:** Minyoung Kwak, Gulsun Erdag, Katie M. Leick, Stefan Bekiranov, Victor H. Engelhard, Craig L. Slingluff

**Affiliations:** 1 Department of Surgery, University of Virginia, P.O. Box 800709, Charlottesville, VA 22908-0709 USA; 2 Carter Center for Immunology Research, University of Virginia, Charlottesville, VA USA; 3 Department of Biochemistry and Molecular Genetics, University of Virginia, Charlottesville, VA USA

**Keywords:** Homing, Chemokines, Integrins, Immune cells, Lymphocytes, Gene expression, Melanoma

## Abstract

**Background:**

Immune cells in the tumor microenvironment have prognostic value. In preclinical models, recruitment and infiltration of these cells depends on immune cell homing (ICH) genes such as chemokines, cell adhesion molecules, and integrins. We hypothesized ICH ligands CXCL9-11 and CCL2-5 would be associated with intratumoral T-cells, while CXCL13 would be more associated with B-cell infiltrates.

**Methods:**

Samples of human melanoma were submitted for gene expression analysis and immune cells identified by immunohistochemistry. Associations between the two were evaluated with unsupervised hierarchical clustering using correlation matrices from Spearman rank tests. Univariate analysis performed Mann–Whitney tests.

**Results:**

For 119 melanoma specimens, analysis of 78 ICH genes revealed association among genes with nonspecific increase of multiple immune cell subsets: CD45^+^, CD8^+^ and CD4^+^ T-cells, CD20^+^ B-cells, CD138^+^ plasma cells, and CD56^+^ NK-cells. ICH genes most associated with these infiltrates included ITGB2, ITGAL, CCL19, CXCL13, plus receptor/ligand pairs CXCL9 and CXCL10 with CXCR3; CCL4 and CCL5 with CCR5. This top ICH gene expression signature was also associated with genes representing immune-activation and effector function. In contrast, CD163^+^ M2-macrophages was weakly associated with a different ICH gene signature.

**Conclusion:**

These data do not support our hypothesis that each immune cell subset is uniquely associated with specific ICH genes. Instead, a larger set of ICH genes identifies melanomas with concordant infiltration of B-cell and T-cell lineages, while CD163^+^ M2-macrophage infiltration suggesting alternate mechanisms for their recruitment. Future studies should explore the extent ICH gene signature contributes to tertiary lymphoid structures or cross-talk between homing pathways.

**Supplementary Information:**

The online version contains supplementary material available at 10.1186/s12967-021-03044-5.

## Background

The density, location, and function of immune cells in the tumor microenvironment (TME), especially CD8^+^ T cells, are prognostic in melanoma [1–6] and other cancers [7–9], and can predict patient responses to immunomodulating therapies [10–13]. Others and we have also shown that the density of intratumoral B cells is associated with prolonged survival in melanoma [5, 14–19]. Individual immune cells can infiltrate among tumor cells, but they can also organize into tertiary lymphoid structures (TLS) in tumor deposits and at their periphery [14]. These TLS resemble small lymph nodes, each containing an organized B cell area, often with features of germinal centers, a T cell area containing CD4^+^ and CD8^+^ T cells and mature dendritic cells, and vasculature expressing peripheral node addressin (PNAd) [14, 20, 21]. The presence of TLS has been associated with favorable outcomes in melanoma and non-small cell lung cancers [22] with higher rates of clinical response to checkpoint blockade therapy of melanoma [23, 24]. The varied distributions of immune cell subsets among melanomas from different patients raise questions about the factors that mediate infiltration of these and other immune subsets.

Homing of immune cells to peripheral tissues, including cancers, is governed by interactions between homing receptors (HRs) on immune cells and homing receptor ligands (HRLs) in tissues, including tumor vasculature. In aggregate, we will refer to these HRs and HRLs as immune cell homing (ICH) molecules. In murine models, effector CD8^+^ T cells expressing HRs α4β1 integrin, the chemokine receptor CXCR3, and E-selectin ligand (ESL), are recruited based on expression of their ligands (VCAM-1, CXCL9/CXCL10/CXCL11, and E selectin (SELE), respectively) in the endothelium of the tumor vasculature [25]. In humans, the chemokines CCL2, CCL3, CCL4, CCL5, CXCL9, and CXCL10 have been associated with increased T cell infiltrates in melanomas, and the same chemokines were confirmed to attract human CD8^+^ T cells in vitro [26]. These genes have been highlighted as crucial for immune rejection of a range of cancers, as part of the “immunologic constant of rejection”, which includes CCL2, CCL5, CXCL9, CXCL10, and VCAM1 in an 18-gene signature [27]. Some of these HRLs are induced by interferon-gamma (IFNγ) in the TME, suggesting a feed-forward loop whereby infiltration by CD8^+^ T cells is followed by their production of IFNγ, which upregulates expression of the HRLs on tumor vasculature, which in turn enhances infiltration by more effector CD8 T cells [25]. B cell infiltration into colorectal and breast cancers requires CXCL13 [4, 17, 28, 29], but to our knowledge, no previous studies have addressed this in melanoma. The trafficking and retention of B cells to secondary lymphoid organs (SLO), however, has been shown to involve CXCL13, CCL19/CCL21, and CXCL12, with their associated HRs: CXCR5, CCR7, and CXCR4, respectively [30]. These ICH pairs may also play a pivotal role in the trafficking of B cells to tertiary lymphoid structures (TLS) of tumors in addition to T cells [21, 31, 32]. ICH genes associated with tumor infiltration by other immune cell subtypes such as macrophages and NK cells have not been well-studied, especially in melanoma.

Melanomas vary widely in the extent of immune infiltrates, from “cold” tumors lacking immune cells to “hot” tumors with diffuse infiltrates [5, 26]. Since the ICH molecules responsible for infiltration are unique to different immune cell subsets as shown in preclinical melanoma models and other solid cancers already described, we expected ICH gene expression in melanomas to be associated with specific infiltrates of immune cell subsets. To test this, we analyzed a wide range of ICH genes and evaluated associations between their expression and infiltrates of immune cell subtypes in melanomas. We hypothesized that the expression of ICH genes would be closely associated with specific immune cell subpopulations found within the tumors. More specifically, we hypothesized that the homing ligands CXCL9-11 and CCL2-5 would be strongly associated with intratumoral T cell infiltrates, whereas CXCL13 would be strongly associated with intratumoral B cell infiltrates.

## Methods

The present report builds on prior data on the density and distribution of immune cell subsets in human melanoma specimens. Specimens previously evaluated by immunohistochemistry (IHC) were sampled for analysis of expression of a panel of immune-related genes.

### Patients, tumor tissue microarrays, and immunohistochemistry

Human melanoma tissue was available from formalin-fixed paraffin-embedded (FFPE) tissue blocks from the archives of the Department of Pathology, University of Virginia. Adult patients diagnosed with melanoma were identified by the Anatomic Pathology Software System (1982–2007). Melanoma specimens with ample tissue and appropriate clinical follow-up were selected for creation of a melanoma tissue microarray (TMA, IRB #10803), as previously described [5]. Areas of tumor from these surgical specimens were identified by a pathologist on hemotoxylin and eosin (H&E) slides (IRB #10598). TMAs were constructed from 3 to 4 cores (1.0 mm diameter) through regions of tumor. Control tissues from normal human liver, spleen, placenta, and kidney were included in each TMA block. TMA tissue sections were evaluated for immune cell infiltrates by immunohistochemistry (IHC) staining using standard protocols. Positive controls included lymph nodes (LN), and, for CD34 antibody, placenta. Negative control slides used PBS instead of primary antibody, with other conditions constant. Immune cells within each core were enumerated by a surgical pathologist [5].

### Immunotype assessments and heterogeneity of findings

We previously reported that melanoma metastases can be categorized into 3 Immunotype Groups based on the extent and patterns of immune cell infiltrates [5]. Quantification of multiple immune cell groups and examples of the three Immunotype Groups in single- and multi-stained melanoma tumors have been previously reported in both human and murine models, with examples illustrated [5, 33, 34]. In each core, intratumoral immune cells were scored as 1 when immune cells (CD45^+^) were absent or sparse (no more than 50 immune cells per 1 mm diameter core); 2 when intratumoral immune cells were present at > 50 CD45^+^ cells/core, but were limited to perivascular cuffing around intratumoral blood vessels; 3 when immune cells were diffusely present among tumor cells in different areas of the core. Mean Immunotype Scores were calculated for each tumor sample, and three Immunotype Groups were created based on CD31^+^ endothelial and CD45^+^ immune cell distribution: Immunotype A for scores < 1.75, Immunotype B for 1.75–2.4, and Immunotype C for >  = 2.5. Additional details have been reported [5].

Triplicate or quadruplicate cores from each tumor sample were evaluated for homogeneity. When the Immunotype Scores were consistent across all three replicates, these samples are considered homogeneous for immunotype pattern. However, when they were not all consistent, these samples are considered heterogeneous for immunotype pattern.

### Quantitative nuclease protection assay

The same FFPE tissue blocks analyzed by IHC were also submitted for gene expression analysis of homing genes and genes related immune cell subtypes (IRB #17816). Five micrometer sections of FFPE tumor specimens were sent for gene analysis to HTG Molecular (HTG Molecular, Tucson, AZ). The HTG EdgeSeq Immuno-Oncology Assay was used for gene expression analysis, which included 558 probes with 15 housekeeper genes, 5 negative and 4 positive processor controls. For this assay, functional DNA Nuclease Protection Probes (NPPs) are flanked by universal wing sequences that are hybridized to the target RNAs. Universal DNA wingmen probes are hybridized to the wings to prevent S1 nuclease digestion. S1 nuclease is added to digest excess non-hybridized RNA and DNA probes. This reaction then results in a stoichiometric quantity of NPPs:RNA hetero-duplexes of interest. Heat denaturization releases the protection probe allowing for enumeration by the Illumina NextSeq™ sequencing platform. Gene expression was standardized through a procedure that log-transformed Counts Per Million (CPM) and adjusted for total reads within a sample. The immune cell homing (ICH) genes included in the analysis were all chemokines, integrins, selectins and Ig superfamily adhesion molecules, and their receptors, along with other molecules previously found to be involved with immune cell chemotaxis and/or cell adhesion as available through the Immuno–Oncology Assay (Additional file 1: Table S1).

### Statistics

Heatmaps were generated using unsupervised hierarchical clustering with Spearman correlation distance metric and Ward clustering method (“Ward.D” method using *hclust* in R, R Core Team). Statistical comparisons between two subgroups and multiple subgroups were performed using the Mann–Whitney and Kruskall–Wallis test, respectively. Correlation coefficients (r values) were performed using the Spearman rank test. Thresholds for high and low gene expression or immune cell and immune marker subgroups were determined by median values. Statistical analysis was performed using Prism 8.0 (Graphpad Software Inc., San Diego, CA), and R (R Core Team). P values < 0.05 were considered significant.

## Results

### A group of immune cell homing genes is associated with Immunotype Score

**Fig. 1:**
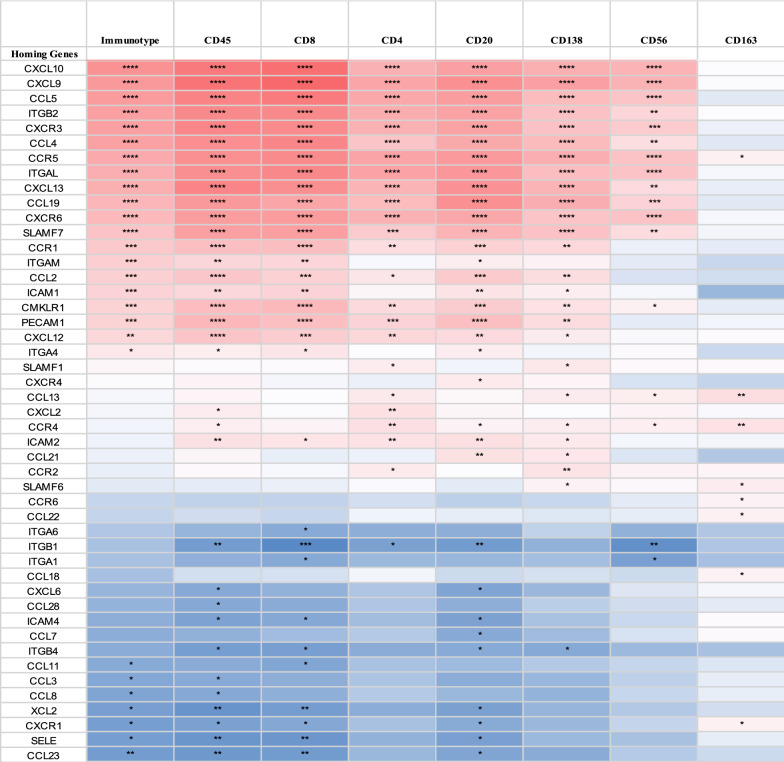
ICH genes associated with Immunotype Group and immune cell densities (per mm^2^) in melanomas. Heat map of correlation coefficients (r values) shown in red represent strong positive correlations, blue represents weaker correlations. P values for each correlation coefficient are shown within each cell; p value < 0.05 (*), < 0.01 (**), < 0.001 (***), < 0.0001 (****). Blank cells had p values > 0.05

**Fig. 2:**
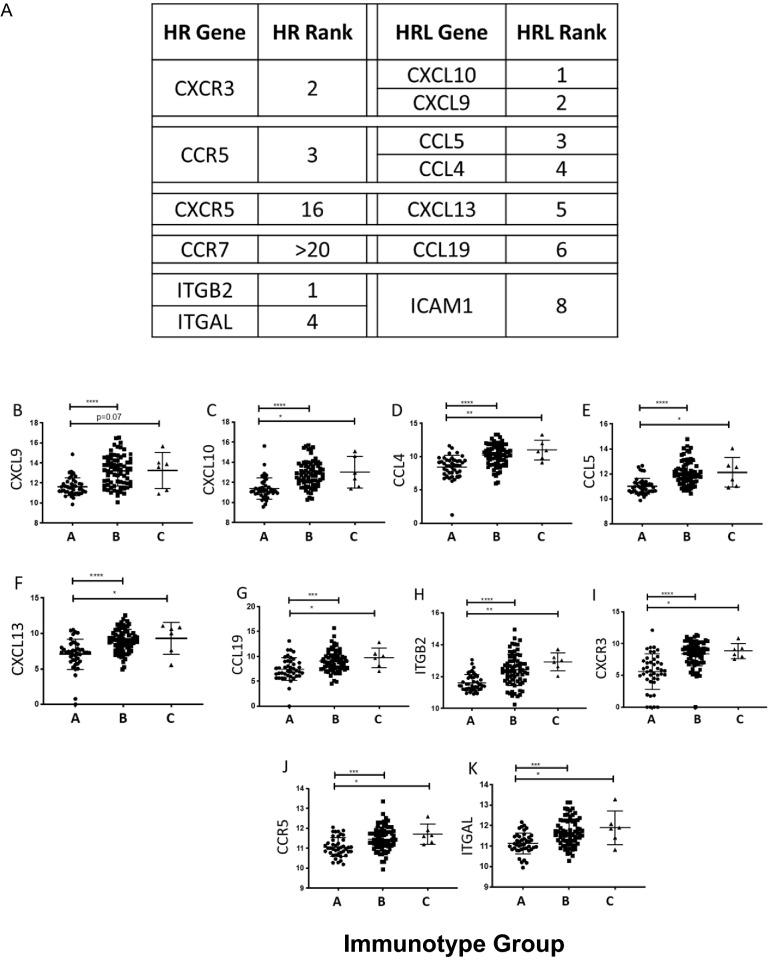
Top ICH genes associated with Immunotype. **A** Top ICH genes are shown as homing receptors (HR) with their associated ligands (HRL). The rank column describes the rank in descending association (based on r value) between the ICH gene and the Immunotype Score. **B-K** ICH gene expression for the Top HRLs and Top HRs for each Immunotype (**A**, **B**, and **C**). The X axis is Immunotype Groups based on the Immunotype Score. Y axis is ICH gene expression level (as normalized log of the counts per million (CPM)). Bars show mean and standard error of the mean. Statistical analysis based on Kruskall-Wallis test. P value < 0.05 (*), < 0.01 (**), < 0.001 (***), < 0.0001 (****)

A total of 119 tumor specimens were included in the study that had both immune cell populations by IHC and gene expression data from the same FFPE block. This included both primary (n = 7, 6%) and metastatic (n = 112, 94.1%) melanoma tumors. No duplicate primary or metastatic tumor specimens were represented. In order to categorize the patterns of intratumoral immune cell populations among the 119 tumors, we used the Immunotype Score from a previous analysis that was based on CD45^+^ immune cell density and distribution relative to the tumor and its proximity to intratumoral vasculature [5]. The Immunotype Score is a continuous variable from 1.0 to 3.0 and used to categorize these values into Immunotype Groups, which are also associated with survival: Immunotype A tumors have few or no infiltrating immune cells (Immunotype Score < 1.75), Immunotype B tumors have immune infiltrates that are limited to perivascular regions (Immunotype Score between 1.75–2.4), and Immunotype C tumors have diffuse immune cell infiltrates (Immunotype Score > 2.4) [5]. Survival is least favorable for Immunotype A melanomas and best for Immunotype C. Tumors included in the study included 35% Immunotype A (n = 43), 60% Immunotype B (n = 71), and 5% Immunotype C (n = 6). Of the 78 total ICH genes evaluated, only 20 (25.6%) ICH genes were significantly and positively associated with the Immunotype Score (p < 0.05, Fig. 1, Additional file 1: Table S1). These included 12 ICH genes with p < 0.0001 and r > 0.35 (CXCL10, CXCL9, CCL5, ITGB2, CXCR3, CCL4, CCR5, ITGAL, CXCL13, CCL19, CXCR6, SLAMF7), plus 8 ICH genes with p values between 0.016—0.0002 and r values between 0.22 and 0.34 (CCR1, ITGAM, CCL2, ICAM1, CMKLR1, PECAM1, CXCL12, ITGA4, SLAMF1). To minimize risk of false discovery, we focused on the top 10 ICH genes (“Top ICH genes”) whose expression was most significantly associated with Immunotype score (all p < 0.0001, and r = 0.41–0.56): CXCL10, CXCL9, CCL5, ITGB2, CXCR3, CCL4, CCR5, ITGAL, CXCL13, CCL19, listed in order of descending r values. We will refer to the HRLs in this list as “Top HRLs” (CXCL10, CXCL9, CCL5, CCL4, CXCL13, and CCL19) and the HRs in this list as the “Top HRs” (ITGB2, CXCR3, CCR5, and ITGAL). Four of these Top HRLs are recognized by 2 of the Top HRs and represent ligand-receptor pairs that have been previously identified as crucial mediators of T and B cell trafficking in melanoma: CXCL10 and CXCL9 are ligands for CXCR3; and CCL4 and CCL5 are ligands for CCR5 (Fig. 2A). The other ICH genes within the Top HRL and Top HR molecules paired with genes that ranked lower (Fig. 2A). The increasing levels of expression of these genes with Immunotype Score and Immunotype Group are shown in Fig. 2B-K and confirm significant increases in gene expression across the Immunotypes from A to C.

### Associations between ICH genes and Immunotype Score for metastatic vs primary tumors and for heterogeneous tumors.

The tumors included in this study consisted of metastatic lesions from skin/subcutaneous sites (n = 64, 57%), lymph nodes (n = 41, 37%), and intra-abdominal tissues (n = 7, 6%), as well as a small number of primary melanomas. To assess whether the associations found between Immunotype Scores and Top ICH genes were present for each of these metastatic sites and primary melanomas, we evaluated those associations with three representative HRL genes from the Top HRL group that are each recognized by different receptors: CXCL9, CCL5, and CXCL13. We also evaluated the association of PTPRC gene (representing CD45^+^ immune cells [35] expression with Immunotype Scores in order to show these associations as compared to representative HRL genes. Expression levels of these representative Top HRL genes were significantly and positively associated with Immunotype Scores for all metastatic tumors (r = 0.46–0.52, p < 0.001), as well as for those in skin/subcutaneous and lymph node sites (r = 0.42–0.58, p < 0.0001 to 0.009) (Table 1). The associations of Immunotype Score with the representative Top HRL were generally stronger than associations with the CD45^+^ immune cell gene, PTPRC, in these same groups. There were only 7 primary melanomas which may explain lack of significant associations in that small subset. Accordingly, there were also only 7 metastatic tumors to small bowel and other abdominal sites, and r values were found to be not significant despite showing relatively strong correlations (r = 0.54–0.62, p = 0.15–0.21) other than CCL5 that had the highest significant r value among all evaluated subsets (r = 0.84, p = 0.024, Table 1). Therefore, the representative Top ICH genes had stronger associations with the Immunotype Score compared to an immune cell marker gene, even in different tumor subsets included in our analysis.

**Table 1:** Associations of Immunotype Scores and immune cell homing (ICH) gene expression levels for tumor subsets. Correlation coefficients (r values) calculated by the Spearman rank test. PTPRC gene used to represent CD45^+^ immune cells for comparison

	N (%)	PTPRC (CD45)		CXCL9		CCL5		CXCL13	
		r value (95% CI)	p value	r value (95% CI)	p value	r value (95% CI)	p value	r value (95% CI)	p value
All Tumors	119	0.32 (0.14–0.47)	0.0005	0.53 (0.38–0.65)	< 0.0001	0.52 (0.37–0.64)	< 0.0001	0.44 (0.28–0.58)	< 0.0001
Tumor type									
Primary	7 (6%)	− 0.39	0.38	0.77	0.06	0.56	0.2	0.17	0.73
Metastatic	112 (94%)	0.32 (0.14–0.48)	0.0006	0.52 (0.36–0.65)	< 0.0001	0.51 (0.36–0.64)	< 0.0001	0.46 (0.29–0.6)	< 0.0001
Metastatic sites									
Skin/subcutaneous	64 (57%)	0.22 (− 0.04–0.45)	0.08	0.52 (0.3–0.68)	< 0.0001	0.58 (0.38–0.72)	< 0.0001	0.42 (0.19–0.61)	0.0005
Lymph Node	41 (37%)	0.39 (0.08–0.63)	0.01	0.46 (0.16–0.68)	0.003	0.4 (0.1–0.64)	0.009	0.48 (0.19–0.69)	0.001
Abdomen	7 (6%)	0.54	0.21	0.62	0.15	0.84	0.024	0.62	0.15
Tumors with heterogeneity in immune infiltrates									
Heterogenous TMA Cores	7 (42%)	0.48 (0.23–0.68)	0.0004	0.52 (0.27–0.7)	0.0001	0.55 (0.31–0.72)	< 0.0001	0.45 (0.18–0.65)	0.001

*95% CI* 95% confidence interval, *TMA* tissue microarray

Next, we determined if this same effect is also true for those tumors for which Immunotype Scores differed among the different core samples in the tissue microarray (TMA) cores analyzed by IHC. Immunotype Scores had been defined for each of 3–4 core tissue samples in a TMA for each tumor, and were identical for each core for 58% of the tumors (homogeneous tumors), but varied among different cores for 50 (42%) tumors (heterogeneous cores). This latter group of heterogenous tumors still had significant associations between the average Immunotype Score and Top HRL gene expression (r = 0.45–0.55, p < 0.0001–0.001, Table 1).

### Association of ICH gene expression with density of immune cell subsets.

**Fig. 3:**
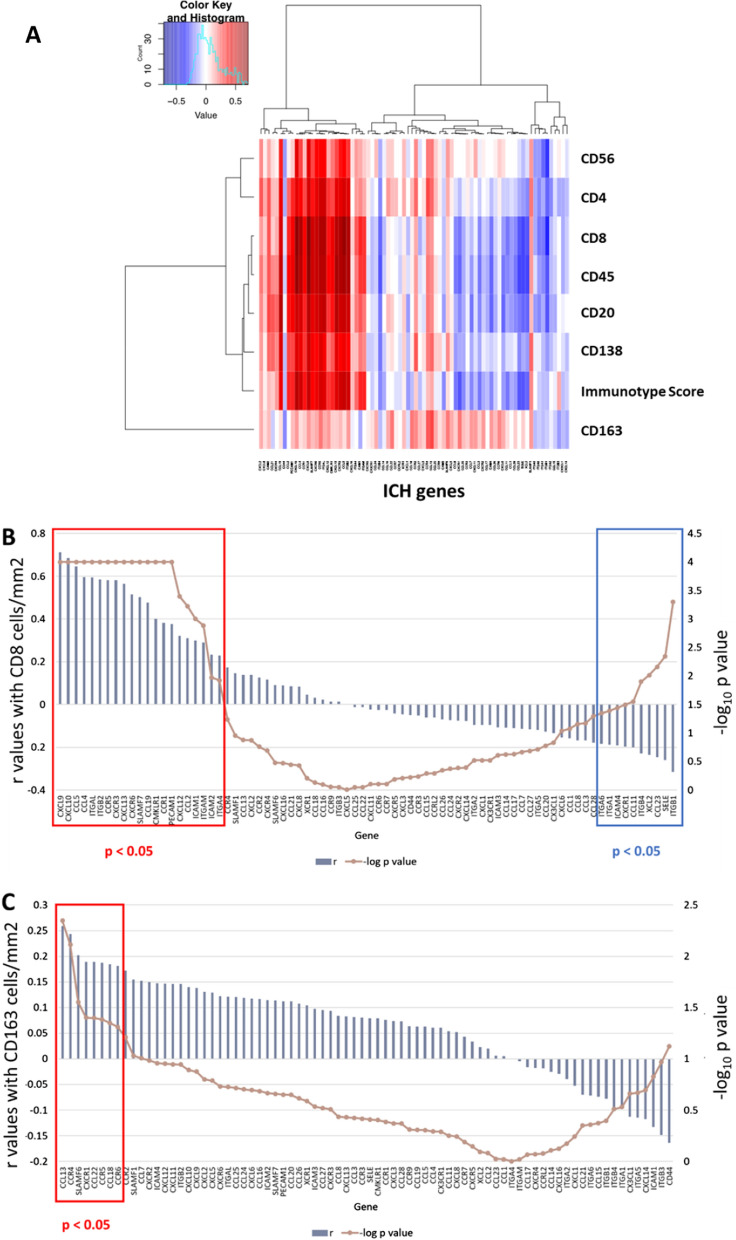
**A** Unsupervised hierarchical clustering based on the correlation matrix between immune cell homing (ICH) gene expression levels and different immune cell subtypes. The correlation matrix of r values was calculated by the Spearman rank test and included ICH gene expression profiles standardized by counts per million on the x axis, and densities of intratumoral immune cell subtypes determined by immunohistochemistry by the Immunotype Score on the y axis using Spearman ward d2. **B**, **C** Graphs showing the r and p values between CD8^+^ (**B**) and CD163^+^ (**C**) cell densities and ICH gene expression levels using a line to represent p values in log10 form for each data point. Red represents higher r values. Blue represents lower r values

Having identified a set of ICH genes whose expression was highly associated with Immunotype Score, we next determined whether distinct subsets of ICH genes were associated with intratumoral density of CD45^+^ cells along with immune cell subsets including CD8^+^ T cells, CD4^+^ T cells, CD20^+^ B cells, CD138^+^ plasma cells, CD56^+^ natural killer-NK cells, and CD163^+^ Type 2 macrophages/monocytes from TMA cores analyzed by IHC on the same tumor specimens as the ICH gene assay. We created an unsupervised heat map of the correlation matrix between the density of each immune cell subtype and ICH gene expression (Fig. 3A). Unsupervised hierarchical clustering showed 3 primary clusters: the largest and central cluster included CD45^+^, CD8^+^, CD20^+^, CD138^+^, and Immunotype Score. This demonstrates a common subset of ICH genes that were consistently associated with each of these cell types and Immunotype Score. This was distinct from a cluster including CD56^+^ and CD4^+^cells, but the most dramatic separation was between all of these cell subtypes and CD163^+^ cells (Fig. 3A).

Within these primary clusters, the common subset of associated ICH genes includes the same Top ICH genes associated most strongly with Immunotype Score. These genes also had the strongest associations for all immune cell subtypes except for CD163^+^ cells (Figs. 1 and 3A, Additional file 1: Table S1). The Top ICH genes had highly significant and similar associations within the primary cluster that included intratumoral densities of CD45^+^ (r = 0.53–0.67, all p < 0.0001), CD8^+^ T cells (r = 0.48–0.71, all p < 0.0001, Fig. 3B), CD20^+^ B cells (r = 0.47–0.56, all p < 0.0001), and CD138^+^ cells (0.37–0.5, all p < 0.0001, Additional file 1: Table S1). The same Top ICH genes also had the strongest r values in the cluster that included CD4^+^ T cells (r = 0.36–0.49, all p < 0.0001), and to a lesser extent with CD56^+^ cells (r = 0.26–0.43, p < 0.001–0.005, Fig. 1). Thus, a shared set of ICH genes are associated with high densities of intratumoral infiltrates with CD8^+^ and CD4^+^ T cells, B cells, plasma cells, and NK cells.

In contrast, densities of tumor-infiltrating CD163^+^ macrophages/monocyte were weakly (r = 0.19, p = 0.04) associated with only one of the top ten ICH genes (CCR5). The other ICH genes most significantly associated with CD163^+^ counts were CCL13 and CCR4 (r = 0.24–0.26, p < 0.008, and 5 other ICH genes (SLAMF6, CXCR1, CCL22, CCL18, CCR6) that had weaker associations but still significantly associated with high CD163^+^ cell densities but not with any of the other cell types (Figs. 1 and 3C, Additional file 1: Table S1). Therefore, the subset of ICH genes that have higher but nonspecific associations with multiple intratumoral immune cell types (CD8^+^ and CD4^+^ T cells, B cells, plasma cells, and NK cells) are not significant with CD163^+^ macrophages/monocyte.

### Overexpression of the Top ICH genes associated with higher intratumoral populations of several immune cell subtypes

**Fig. 4:**
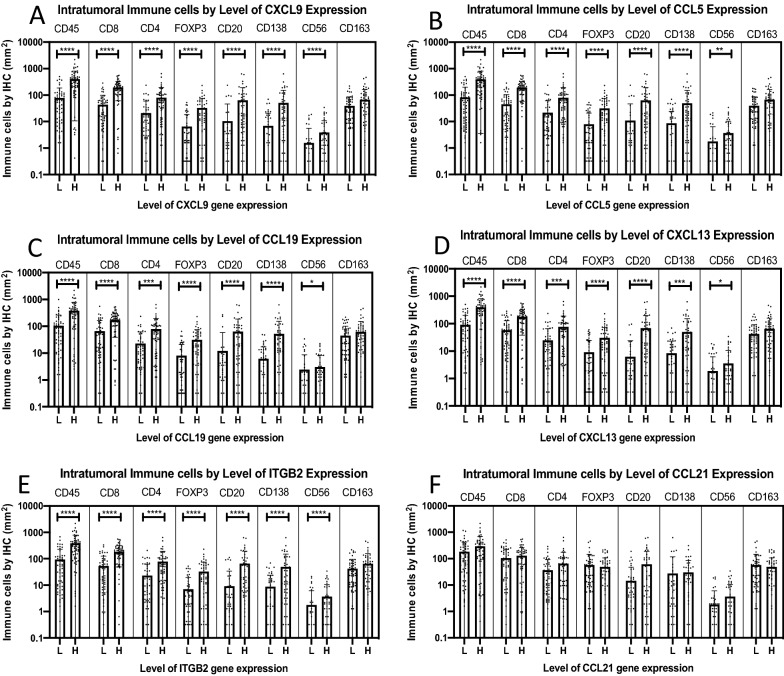
Immune cell populations by IHC based on expression level of top homing genes. X axis is low (L) and high (H) levels of gene expression: **A** CXCL9, **B** CCL5, **C** CCL19, **D** CXCL13, **E** ITGB2, and **F** CCL21. Y axis is enumerated immune cells by IHC per mm^2^. Bars showing mean and standard error of the mean. Statistical analysis based on Mann–Whitney test. P value < 0.05 (*), < 0.01 (**), < 0.001 (***), < 0.0001 (****)

To understand whether associations between ICH gene expression and immune cell infiltrates also translate into significant differences when dichotomizing above and below median ICH gene expression levels, we evaluated intratumoral immune cell densities based on high vs low expression of five representative Top ICH genes (CXCL9, CCL5, CCL19, CXCL13, and ITGB2). We expanded the analysis to include FoxP3^+^ cells in addition to those assessed above. Tumors with high ICH gene expression had significantly higher densities of CD45^+^, CD8^+^ and CD4^+^ T cells, FoxP3^+^ cells, CD20^+^ and CD138^+^ cells (p < 0.01, Fig. 4A–E). Thus, despite less significant associations of Top ICH gene expression with CD4 T cell densities, in this dichotomized analysis, CD4 densities were significantly higher with high ICH expression. Even differences in CD56^+^ cell densities were significant for high vs low ICH gene expression for all 5 of these ICH genes, but were much less significant (0.01 < p < 0.05) for CCL19 and CXCL13 than differences for the other cell subsets (p < 0.001) as shown in Fig. 4A–E. On the other hand, there was no difference in CD163^+^ cell densities with high vs low expression levels of these five top ICH genes (Fig. 4A–E).

Interestingly, CD20^+^ and CD138^+^ cell densities were associated uniquely with one ICH gene, CCL21, where the level of gene expression was not significantly associated with Immunotype Score or with any other individual immune cell subtype (Fig. 1, Additional file 1: Table S1). CCL21 overexpression, however, was not associated with any intratumoral cell density, even for CD20^+^ and CD138^+^ cells (Fig. 4F). Therefore, the overexpression of only the Top ICH genes drove the nonspecific increase of multiple intratumoral cell types except for CD163^+^ Type 2 macrophages/monocytes. Even though other ICH genes outside of the Top ICH gene group such as CCL21 had significant correlations with more specific immune cells subsets (CD20^+^ and CD138^+^), CCL21 overexpression did not also lead to increases in intratumoral CD20^+^ or CD138^+^ cell densities.

### Overexpression of pro-inflammatory gene markers led to higher levels of ICH gene expression

**Fig. 5:**
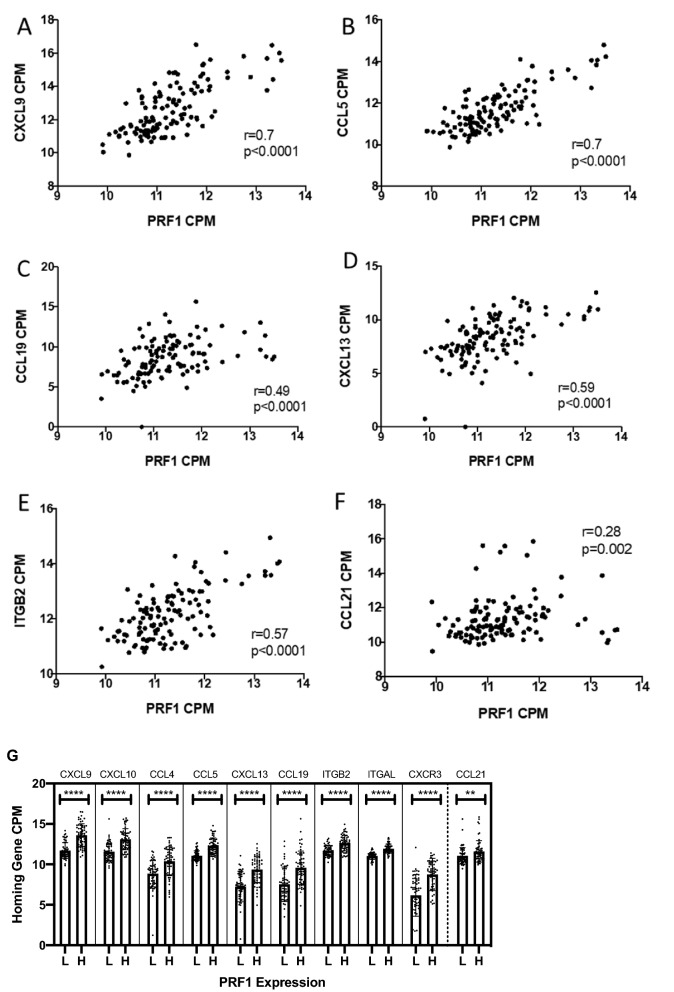
Associations of PRF1 gene expression with expression of selected ICH genes. Scatter plots of **A** CXCL9, **B** CCL5, **C** CCL19, **D** CXCL13, **E** ITGB2, **F** CCL21 with PRF1 gene expression are shown, along with their associated r and p values. **G** Tumors with low (L) and high (H) expression of PRF1 are grouped, showing expression levels for the 10 Top ICH genes, and CCL21 for comparison. P value < 0.05 (*), < 0.01 (**), < 0.001 (***), < 0.0001 (****)

One possible explanation for concordant overexpression of Top ICH genes in association with increased densities of multiple immune cell subtypes is that these ICH genes are induced by pro-inflammatory cytokines in the TME, such as interferon-gamma (IFNγ). An IFNγ signature includes IFNG, PRF1, GZMA, STAT1, IRF1 among others, and also includes the chemokines CXCL9, CXCL10, and CXCL11 [36]. We did not find strong positive associations between expression of the Top ICH genes and IFNG, nor with type I interferons such as IFNA-family or IFNB1 (data not shown). On the other hand, there were highly significant associations with other known pro-inflammatory gene markers. PRF1 gene expression was strongly associated with the top representative ICH genes (r = 0.5–0.7, all p < 0.0001, Fig. 5A–F). Tumors with high expression of PRF1 also had higher expression of the Top ICH genes (all p < 0.0001, Fig. 5G). Highly significant associations with CXCL9, CCL5, CCL19, CXCL13, and ITGB2 were also found with the granzyme A gene (GZMA, r = 0.34–0.54, p < 0.001–0.0002, data not shown), STAT1:STAT3 ratio (r = 0.4–0.71, all p < 0.0001, Additional file 2: Figure S1), and IRF1 (r = 0.21–0.51, p < 0.0001–0.02, Additional file 2: Figure S2). The pattern of expression for CCL21 was again unique compared to the other Top ICH genes, and showed weaker (r = 0.28, p = 0.002, Fig. 5F) associations with PRF1 expression, and was not significantly associated with STAT1:STAT3 ratio or with IRF1 expression (Additional file 2: Figure S1F-G, Figure S2F-G). These data suggest that melanomas with dense infiltrates of T and B cells are characterized by concordant overexpression of multiple ICH genes as well as multiple other genes associated with immune signatures.

## Discussion

Concordant overexpression of a set of immune cell homing genes was significantly associated with patterns of intratumoral immune cell infiltration (Immunotype) and with multiple immune cell subtypes. We evaluated 78 ICH genes and identified 10 ICH genes with the highest associations with increased intratumoral densities of CD45^+^, CD8^+^ and CD4^+^ T cells, CD20^+^ B cells, CD138^+^ plasma cells, and CD56^+^ NK cells. These ICH genes have been reported to support T cell recruitment through ICAM-1 (ITGB2, ITGAL), to support recruitment and retention of activated T cells (CXCR3, CXCL9, CXCL10), to support recruitment of naïve T cells (CCL19), and to support B cell recruitment and retention (CXCL13). Overall, an unexpected finding was that the top ICH genes were commonly concordantly expressed, suggesting that although selected ICH molecules may govern homing and infiltration of each immune cell subset, they are commonly co-expressed in human melanomas.

For some HR/HRL pairs in the top ICH group, overexpression of both the HR and its ligand was observed; however, for some of the top ICH genes, their corresponding receptor or ligand was not included in the list. Part of this discrepancy may be due to the downregulation of expression of some HRs after binding to their ligands [37, 38].

CD163^+^ (Type 2 macrophages/monocytes) cells were the only immune cell subtype included in our analysis whose intratumoral densities were not associated with the top 10 ICH genes. Instead, a smaller, separate group of ICH genes were found to have weakly significant associations with CD163^+^ cell densities. This finding is consistent with our prior findings that CD163^+^ cells were present at similar densities in all melanomas, regardless of whether other immune cells were highly represented [5]. Together, these data suggest that factors controlling homing and retention of CD163^+^ macrophages in melanoma differ from those governing infiltration of lymphocytes.

Our study is limited by the fact that the gene expression assay did not include RNA probes for all ICH genes of interest, such as VCAM-1, MADCAM-1, and L-selectin with their paired receptors, and so it is possible that other homing genes may associate with more specific immune cell subtypes. However, the expression of these homing molecules are also stimulated by inflammation [25, 39] and so may yield similar results. This study is also limited by its retrospective design and the TMA IHC analysis of tumors that did not include further phenotypic subtyping of immune cells so that differences in naïve versus activated immune cell recruitment is unknown.

This current study focused on immune cells that successfully infiltrated melanomas. We did not address homing receptor expression on circulating T cells, as studies by us and by others have previously evaluated them in the setting of melanoma. CXCR3 expression on antigen-experienced peripheral CD8^+^ T cells has been associated with prolonged survival in patients with stage III melanoma, and CCR4 was highly co-expressed with CXCR3 [40]. Most circulating CD8^+^ T cells induced by a peptide vaccine, administered with an incomplete Freund’s adjuvant, also expressed CXCR3, and when GM-CSF was included in the vaccine as well, there was enhanced expression of both CXCR3 and cutaneous leukocyte antigen (CLA), both of which have been implicated in T cell homing [41]. Further, those T cells co-expressing CXCR3 and CLA expressed high levels of interferon-gamma, Tbet, and IL-12 receptor beta[41]. Also, T cells engineered to express CXCR2 [42, 43] or CXCR1 [44] have greater capacity for homing and infiltration of tumor in murine models. Another study evaluated expression patters of nine homing receptors by circulating T cells in patients with metastatic melanoma and found that early expansion of circulating memory CD8^+^ T cells expressing CLA predicted disease control after blockade of CTLA4 and that altered expression of selected chemokines was associated with metastatic disease at corresponding tissue sites [45]. These studies highlight the clinical importance of homing receptors on T cells in order to home adequately to the appropriate tumor-associated vasculature and, thus, to enable tumor infiltration. Our study suggests that once T cells have infiltrated tumors, there appears to be concordant enhancement of expression of a number of other homing molecules, as well. This may reflect concordant enhancement of multiple chemokine receptors at the time of T cell activation, as has been observed in murine studies [46], or it may reflect changes once the T cells have entered the tumor microenvironment. Based on other murine studies, once T cells infiltrate and respond to tumor antigens, the IFN-gamma they produce can enhance expression of multiple homing receptor ligands, which in turn enhances further recruitment of other T cells [25]. There is a need for greater understanding of the relative contribution of these mechanisms to the findings in the present manuscript of highly concordant overexpression of multiple homing receptors and their ligands in human melanoma metastases.

Prior work has shown that ICH genes for CD8^+^ T cells [25–27] differ from those for B cells [4, 17, 28, 29], and for other lymphocyte subsets as previously mentioned, but we are not aware of prior work that has directly assessed whether each of these ICH genes is associated with multiple immune cell subsets in melanomas. Our finding that the top ICH genes were strongly associated with intratumoral density of both T cells and B cells is not likely to be due to direct effects of each of those genes on all lymphocyte subsets, but instead likely reflects more complex features of the immune-activated TME. The presence of tertiary lymphoid structures in melanomas has highlighted the fact that a large proportion of human melanomas contain these structures comprised of B cells and T cells, in addition to dendritic cells [14, 24, 47, 48]. The factors controlling creation of TLS remain to be defined, but the frequent presence of TLS in melanomas suggests that concurrent recruitment and retention of T and B cells into melanomas is not likely to be random and that there must be mechanisms to ensure recruitment of both T cells and B cells. The same ICH genes that resulted in higher intratumoral populations of different cell subtypes were also significantly associated with expression of selected pro-inflammatory genes and immune effector genes, many of which have previously been found to associate with TLS [14]. Further studies may elucidate how those genes or other inflammatory cytokines support ICH gene expression in the TME. Despite associations of ICH gene expression with expression of IRF1, PRF1, GZMA, or the ratio of STAT1:STAT3 expression, we found weak or no associations with expression of IFNG or type 1 interferons directly. This might be explained by issues with the RNA primers, or by previous work showing chronic interferon signaling results in negative feedback loops that inhibit the immune response and may induce epigenomic changes that actually inhibit tumoricidal activity [49–51].

A comparable study in colorectal cancer also showed similar non-specificity of inflammation-induced chemokine genes with intratumoral CD45RO, macrophages and NK cells [52]. That study found 4 of the 16 ICH genes included in their hierarchical clustering model (CXCL9, CXCL10, CCL5 and CCR5), among the same chemokines also found in our analysis, were significantly associated with more than one immune cell subtype. As our data show, a possible cause for this result may be due to the corresponding inflammatory TME of those tumors.

## Conclusions

This study does not support our hypothesis that each immune cell subset is uniquely associated with specific ICH genes. Instead, overexpression of a larger set of immune cell homing genes identifies melanomas with concordant infiltration of B-cell and T-cell lineages, while CD163^+^ M2-macrophage infiltration suggest alternate mechanisms for their recruitment. This may be explained by interferon gamma signaling and effector function. Future studies should explore the extent ICH gene signature contributes to tertiary lymphoid structures or cross-talk between homing pathways.

## Supplementary Information

**Additional file 1:Table S1.** Correlation coefficients of all immune cell homing (ICH) genes included in the analysis. Correlation coefficients (r values) were calculated from levels of immune cell homing gene expression and the Immunotype Score, CD45^+^, CD8^+^, CD4^+^, CD20^+^, CD138^+^, CD56^+^, and CD163^+^, as determined by immunohistochemistry. Correlation coefficients sorted in descending order.

**Additional file 2:Figure S1**. Associations of the ratio of STAT1:STAT3 gene expression with expression of selected ICH genes. Scatter plots of **A** CXCL9, **B** CCL5, **C** CCL19, **D** CXCL13, **E** ITGB2, **F** CCL21 with PRF1 gene expression are shown, along with their associated r and p values. **G** Tumors with low (L) and high (H) expression of STAT1:STAT3 are grouped, showing expression levels for the 10 Top ICH genes, and CCL21 for comparison. P value <0.05 (*), <0.01 (**), <0.001 (***), <0.0001 (****). **Figure S2**. Associations of IRF1 gene expression with expression of selected ICH genes. Scatter plots of **A** CXCL9, **B** CCL5, **C** CCL19, **D** CXCL13, **E** ITGB2, **F** CCL21 with PRF1 gene expression are shown, along with their associated r and p values. **G** Tumors with low (L) and high (H) expression of IRF1 are grouped, showing expression levels for the 10 Top ICH genes, and CCL21 for comparison. P value <0.05 (*), <0.01 (**), <0.001 (***), <0.0001 (****).

## Data Availability

The gene expression dataset generated during the current study is available in the NCBI GEO repository, [GEO accession number pending].

## Abbreviations

TME: Tumor microenvironment
HRL: Homing receptor ligand
HR: Homing receptor
ICB: Immune checkpoint blockade
IHC: Immunohistochemistry
ICH: Immune cell homing
IFNγ: Interferon-gamma
SLO: Secondary lymphoid organ
H&E: Hemotoxylin and eosin
FFPE: Formalin-fixed paraffin-embedded
qNPA: Quantitative nuclease protection assay
TMA: Tumor microarray
TLS: Tertiary lymphoid structures
NK: Natural killer
