# Supplementary material for: Associations of immune cell homing gene signatures and infiltrates of lymphocyte subsets in human melanomas: discordance with CD163+ myeloid cell infiltrates
Source: J Transl Med. 2021 Aug 28;19:371. doi: 10.1186/s12967-021-03044-5 (PMC8403429; doi:10.1186/s12967-021-03044-5)

Supplemental Table: Correlation coefficients of all immune cell homing (ICH) genes included in the analysis. Correlation coefficients (r values) were calculated from levels of immune cell homing gene expression and the Immunotype Score, CD45^+^, CD8^+^, CD4^+^, CD20^+^, CD138^+^, CD56^+^, and CD163^+^, as determined by immunohistochemistry. Correlation coefficients sorted in descending order.


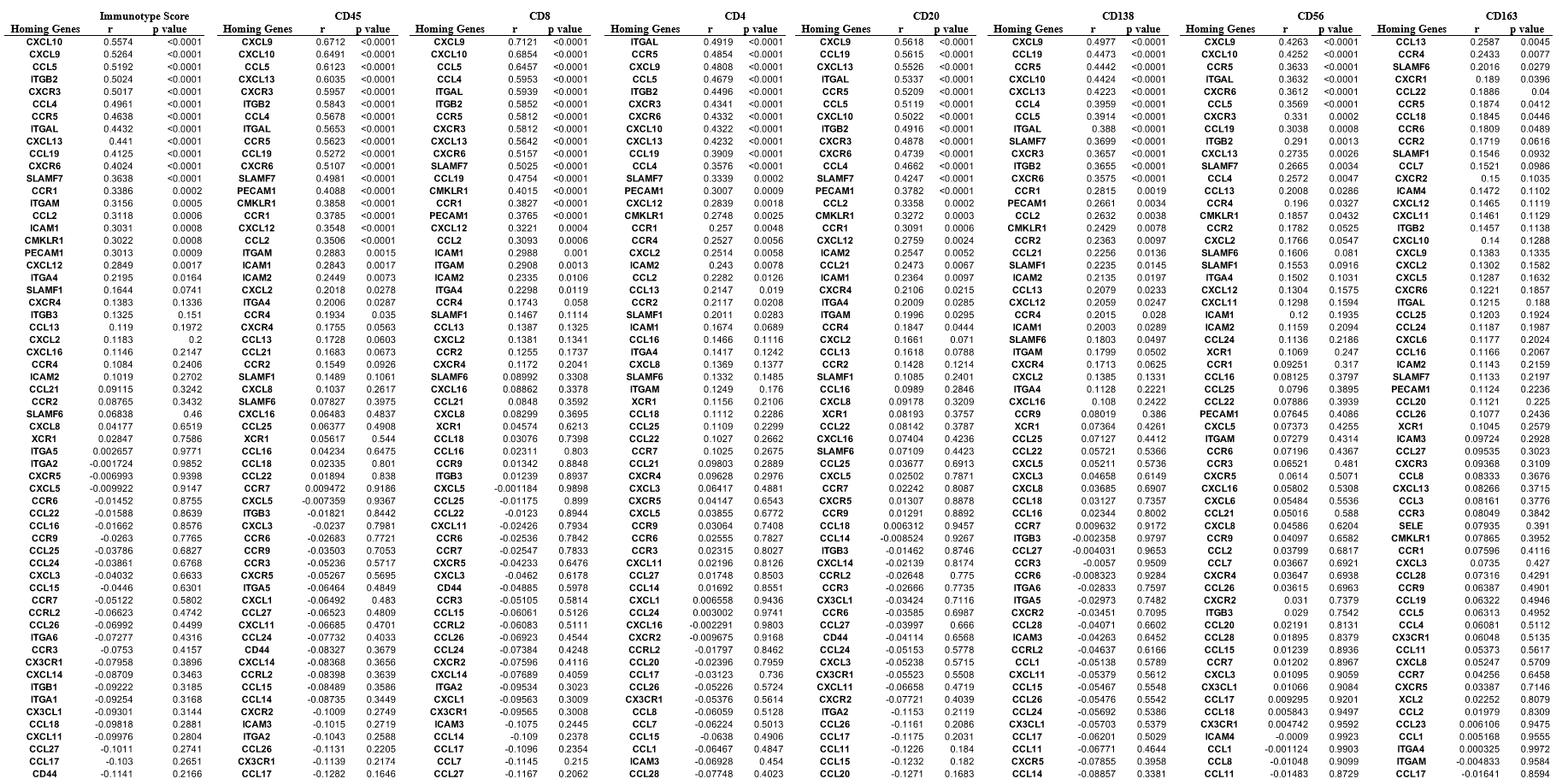


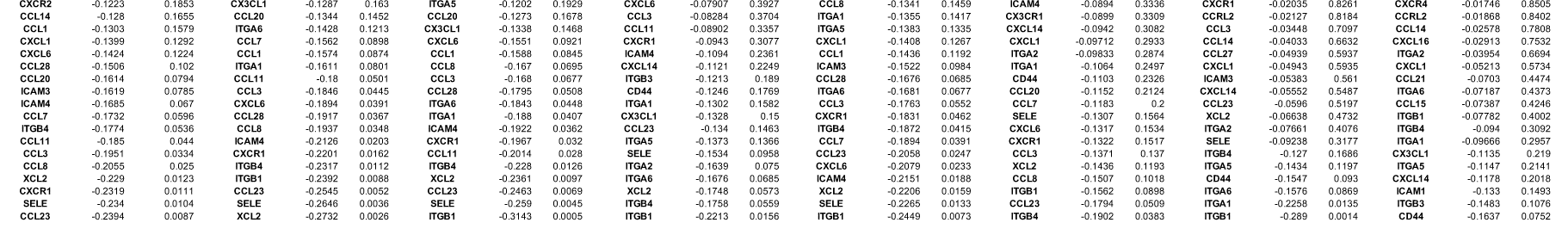

Supplement: Supplementary file 1 — Additional file 1:Table S1. Correlation coefficients of all immune cell homing (ICH) genes included in the analysis. Correlation coefficients (r values) were calculated from levels of immune cell homing gene expression and the Immunotype Score, CD45+, CD8+, CD4+, CD20+, CD138+, CD56+, and CD163+, as determined by immunohistochemistry. Correlation coefficients sorted in descending order. [file 12967_2021_3044_MOESM1_ESM.docx]
