# Supplementary material for: Associations of immune cell homing gene signatures and infiltrates of lymphocyte subsets in human melanomas: discordance with CD163+ myeloid cell infiltrates
Source: J Transl Med. 2021 Aug 28;19:371. doi: 10.1186/s12967-021-03044-5 (PMC8403429; doi:10.1186/s12967-021-03044-5)
Supplement: Supplementary file 2 — Additional file 2:Figure S1. Associations of the ratio of STAT1:STAT3 gene expression with expression of selected ICH genes. Scatter plots of A CXCL9, B CCL5, C CCL19, D CXCL13, E ITGB2, F CCL21 with PRF1 gene expression are shown, along with their associated r and p values. G Tumors with low (L) and high (H) expression of STAT1:STAT3 are grouped, showing expression levels for the 10 Top ICH genes, and CCL21 for comparison. P value <0.05 (*), <0.01 (**), <0.001 (***), <0.0001 (****). Figure S2. Associations of IRF1 gene expression with expression of selected ICH genes. Scatter plots of A CXCL9, B CCL5, C CCL19, D CXCL13, E ITGB2, F CCL21 with PRF1 gene expression are shown, along with their associated r and p values. G Tumors with low (L) and high (H) expression of IRF1 are grouped, showing expression levels for the 10 Top ICH genes, and CCL21 for comparison. P value <0.05 (*), <0.01 (**), <0.001 (***), <0.0001 (****). [file 12967_2021_3044_MOESM2_ESM.docx]

**Supplemental Figure 1**. Associations of the ratio of STAT1:STAT3 gene expression with expression of selected ICH genes. Scatter plots of (**A**) CXCL9, (**B**) CCL5, (**C**) CCL19, (**D**) CXCL13, (**E**) ITGB2, (**F**) CCL21 with PRF1 gene expression are shown, along with their associated r and p values. (**G**) Tumors with low (L) and high (H) expression of STAT1:STAT3 are grouped, showing expression levels for the 10 Top ICH genes, and CCL21 for comparison. P value <0.05 (*), <0.01 (**), <0.001 (***), <0.0001 (****).


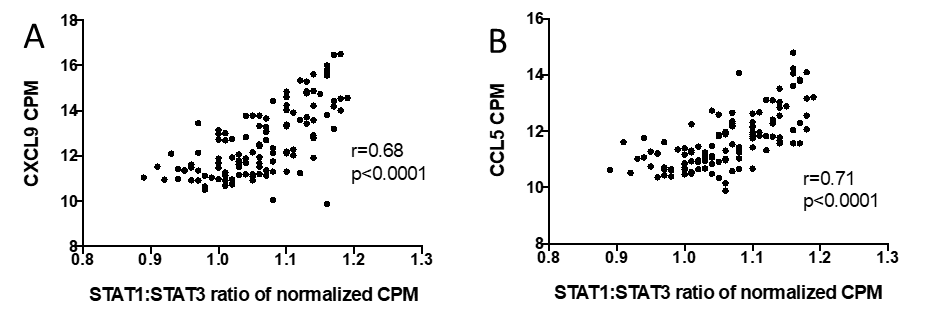


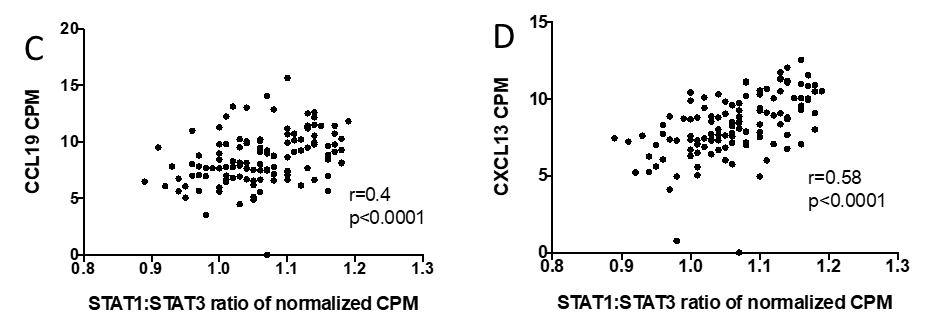


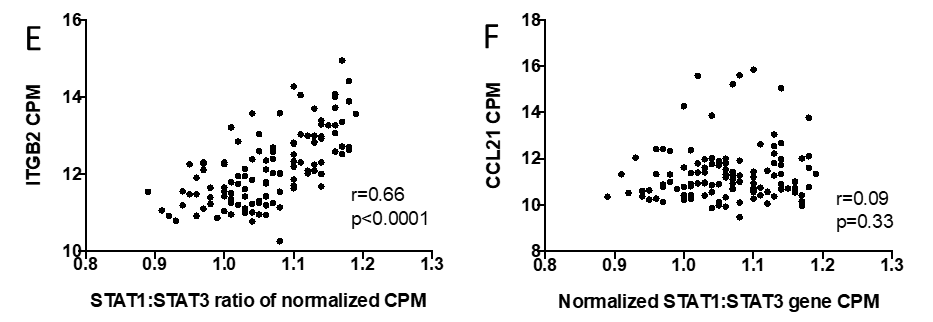


G

**Supplemental Figure 2**. Associations of IRF1 gene expression with expression of selected ICH genes. Scatter plots of (**A**) CXCL9, (**B**) CCL5, (**C**) CCL19, (**D**) CXCL13, (**E**) ITGB2, (**F**) CCL21 with PRF1 gene expression are shown, along with their associated r and p values. (**G**) Tumors with low (L) and high (H) expression of IRF1 are grouped, showing expression levels for the 10 Top ICH genes, and CCL21 for comparison. P value <0.05 (*), <0.01 (**), <0.001 (***), <0.0001 (****).


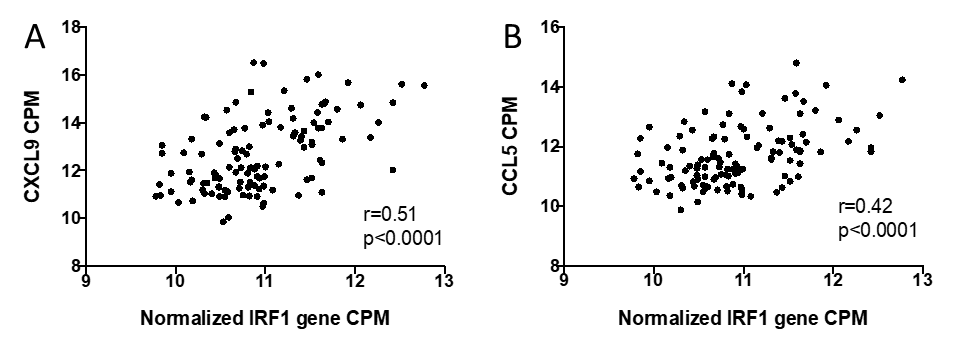


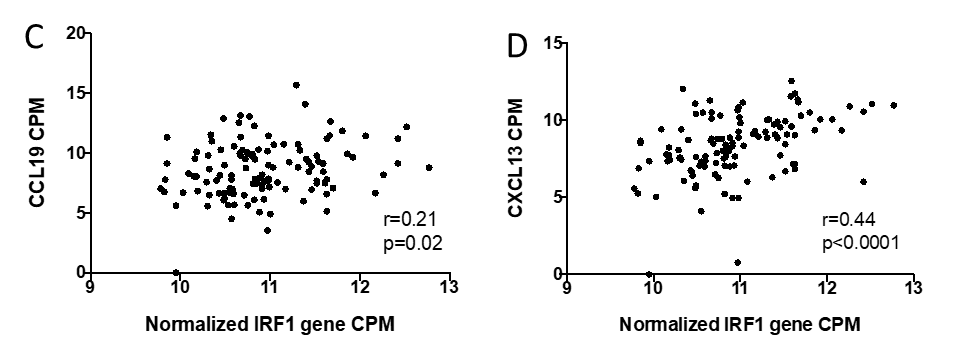


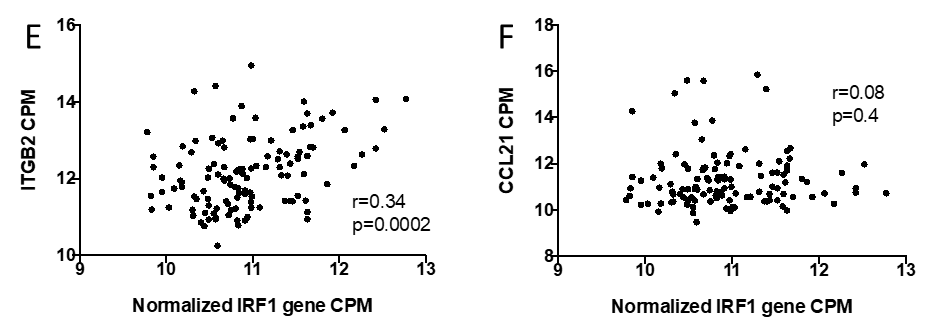


G
